# Identifying Consensus Disease Pathways in Parkinson's Disease Using an Integrative Systems Biology Approach

**DOI:** 10.1371/journal.pone.0016917

**Published:** 2011-02-22

**Authors:** Yvonne J. K. Edwards, Gary W. Beecham, William K. Scott, Sawsan Khuri, Guney Bademci, Demet Tekin, Eden R. Martin, Zhijie Jiang, Deborah C. Mash, Jarlath ffrench-Mullen, Margaret A. Pericak-Vance, Nicholas Tsinoremas, Jeffery M. Vance

**Affiliations:** 1 John P. Hussman Institute for Human Genomics, Miller School of Medicine, University of Miami, Miami, Florida, United States of America; 2 Center for Computational Science, Miller School of Medicine, University of Miami, Miami, Florida, United States of America; 3 Department of Neurology and Molecular and Cellular Pharmacology, Miller School of Medicine, University of Miami, Miami, Florida, United States of America; 4 Gene Logic, Gaithersburg, Maryland, United States of America; University of Michigan, United States of America

## Abstract

Parkinson's disease (PD) has had six genome-wide association studies (GWAS) conducted as well as several gene expression studies. However, only variants in MAPT and SNCA have been consistently replicated. To improve the utility of these approaches, we applied pathway analyses integrating both GWAS and gene expression. The top 5000 SNPs (p<0.01) from a joint analysis of three existing PD GWAS were identified and each assigned to a gene. For gene expression, rather than the traditional comparison of one anatomical region between sets of patients and controls, we identified differentially expressed genes between adjacent Braak regions in each individual and adjusted using average control expression profiles. Over-represented pathways were calculated using a hyper-geometric statistical comparison. An integrated, systems meta-analysis of the over-represented pathways combined the expression and GWAS results using a Fisher's combined probability test. Four of the top seven pathways from each approach were identical. The top three pathways in the meta-analysis, with their corrected p-values, were axonal guidance (p = 2.8E-07), focal adhesion (p = 7.7E-06) and calcium signaling (p = 2.9E-05). These results support that a systems biology (pathway) approach will provide additional insight into the genetic etiology of PD and that these pathways have both biological and statistical support to be important in PD.

## Introduction

Parkinson's disease [PD (OMIM 168600)] is a complex neurodegenerative disease thought to be due to interactions between genetic susceptibility and environmental stressors. Several genome-wide association studies (GWAS) on PD have been performed recently [1]–[5], but only two genes have demonstrated statistical significance and replication across data sets: the Microtubule-Associated Protein Tau (MAPT) and α-synuclein (SNCA). However, many genes have been replicated in these datasets, but after correcting for multiple testing, have not reached genome-wide significance. Thus, additional approaches are needed to maximize the existing genetic data to identify genes and pathways important in developing PD.

A criticism of many of the current GWAS analytical approaches is that they focus primarily on the most significant SNPs occurring in an analysis [6], and that this may not incorporate known biological information that could increase their value. One way to utilize biological information is a pathway-analysis approach, based on the realization that complex diseases are likely affected by many genes contributing in a common pathway [2], [7]–[10].

Another source of genetic data for research in complex genetic disease has been gene expression [11]–[13]. But interpretation of these gene expression results, especially in complex diseases, has potential difficulties. For one, disease risk may well be conferred through means other than gene expression changes, and thus genetic variation important to disease risk may not affect gene expression directly, and not be detected through such studies. Another often cited criticism is that many of the differentially expressed genes between patients and controls may actually be secondary to degenerative changes in patients, and thus have little contribution to the actual disease process.

The classical experimental method employed in gene expression analyses has been to analyze a group of disease cases versus a group of controls. One flaw of this design is that individual sample variations that are secondary to sample handling, agonal state and other general factors can mask or introduce error in the analysis, as the model lacks an internal reference for these effects on gene expression. Further, individuals in late-onset disorders like PD will likely be at different stages of neurodegeneration at death, and thus pooling different brain regions that may be differentially affected from these individuals will introduce more error in the analyses.

We hypothesize that by combining a pathway approach for both GWAS and gene expression we can address many of these concerns and identify those pathways important in the pathogenesis of PD. To reduce error in gene expression, we identified differentially expressed genes between different brain tissues involved in PD in a single individual, rather than comparing average gene expression results from the same anatomical tissue in groups of cases and controls. We then used these data to identify pathways, which were significantly over-represented by differentially expressed genes. Further, a pathway analysis was performed to identify pathways over-represented in the GWAS data. Convincingly, out of 205 potential human pathways listed in the Kyoto Encyclopedia of Genes and Genomes (KEGG), four of the top seven most significant pathways from each approach were identical and meta-analysis demonstrated 10 pathways to be highly significant. These shared pathways from both gene expression and GWAS therefore have both biological and genetic support for their importance in the pathogenesis of PD.

## Results

### Gene Expression Analysis

**Figure 1:**
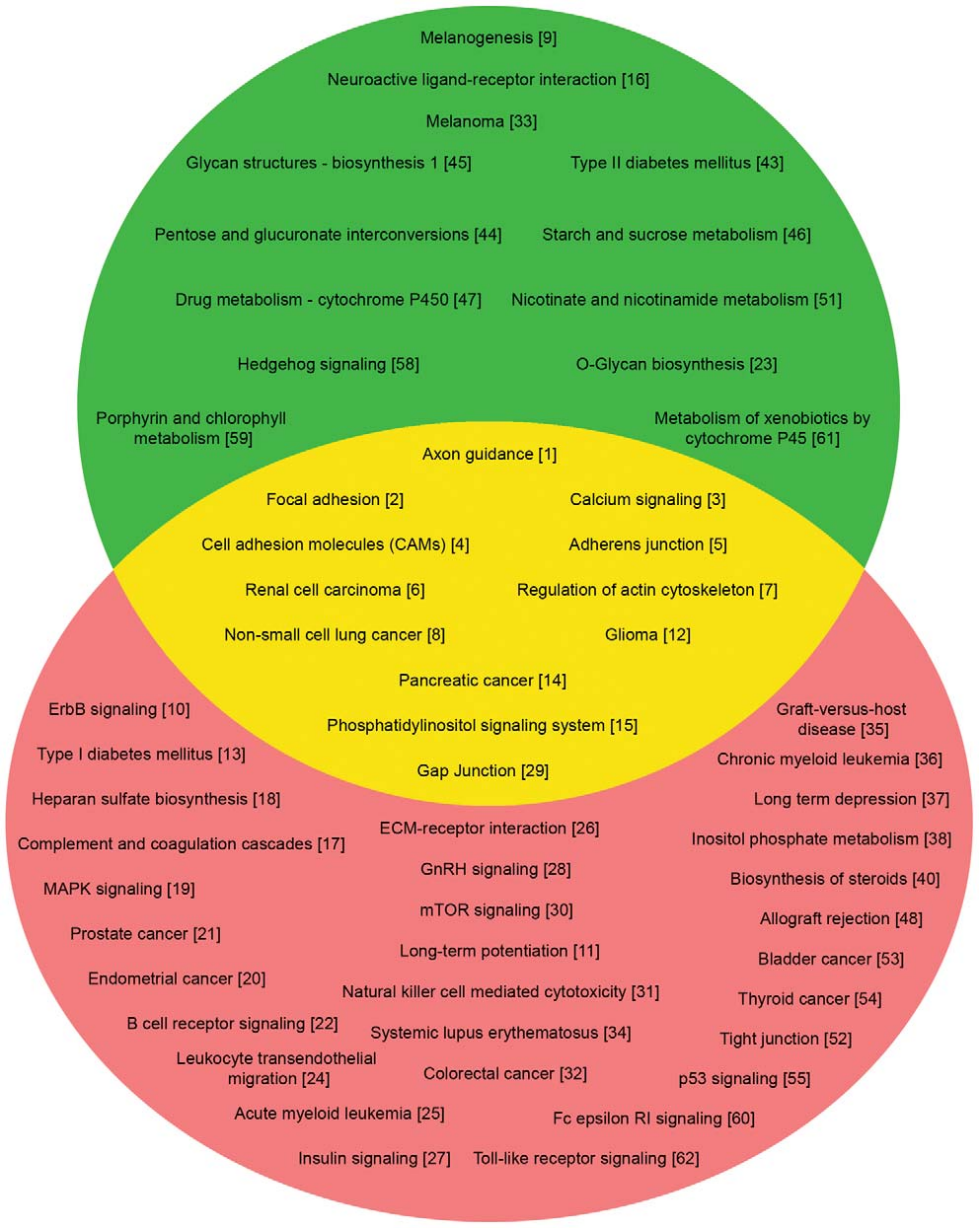
The Venn diagram shows 25 and 42 over-represented pathways in the GWAS (green) and the expression PD (pink) studies respectively. Twelve over-represented pathways common to both PD studies are also shown (yellow). The pathway's rank in the meta-analysis is provided in parenthesis.

Both control and patient subtractions gave a large number of differentially expressed genes between different tissue regions. In controls, this represents the normal functional variation between brain regions. Thus, the average control values were subtracted from each patient sample, normalizing the subtraction results. After the subtraction of the control ratios, a large number of genes remained differentially expressed between subtraction pairs. These were used in the subsequent pathway analysis. 6759 genes are differentially expressed and 42 over-represented pathways were identified (significance level: p≤0.05) (Figure 1).

### GWAS pathway analysis

Twenty-five over-represented pathways at significance level p≤0.05 (Figure 1) were seen in the 5000 SNPs. The 10 most statistically significant over-represented pathways are shown in Table 1. A total of 12 over-represented pathways are common to both the GWAS and the gene expression PD studies (Figure 1). The list of over-represented pathways common for the two PD studies (GWAS or gene expression) does not change if the gene expression data are considered as two sub groups (up–regulated and down-regulated) or merged into a single group (of differentially expressed genes). Additionally, four of the top seven over-represented pathways from each approach (Table 1) were identical; these are: calcium signaling, axon guidance, focal adhesion and adherens junction.

**Table 1:** The top ten over-represented pathways from (a) the GWAS PD study and (b) the gene expression PD study.

(a) GWAS	P value
Melanogenesis	6.51E-05
Axon guidance	8.05E-05
Cell adhesion molecules (CAMs)	9.62E-05
Neuroactive ligand-receptor interaction	3.70E-04
Adherens junction	6.95E-04
Focal Adhesion	8.56E-04
Calcium Signalling pathway	1.94E-03
Pentose and glucuronate interconversions	7.06E-03
Starch and sucrose metabolism	7.90E-03
Drug metabolism - cytochrome P450	8.11E-03

(b) Gene expression	
Axon guidance	7.00E-07
Focal adhesion	2.11E-06
Calcium signaling pathway	3.70E-06
Renal cell carcinoma	9.18E-06
Regulation of actin cytoskeleton	2.24E-05
Adherens junction	9.64E-05
ErbB signaling pathway	1.03E-04
Type I diabetes mellitus	1.13E-04
Long-term potentiation	4.37E-04
Non-small cell lung cancer	4.84E-04

The pathways are presented in the decreasing order of significance.

### Meta-Analysis of Over-represented Pathways in PD

A meta-analysis of over-represented pathways was developed and performed to identify statistical significance in the combined GWAS and gene expression PD study. For the gene expression data, the up–regulated and down-regulated genes are merged into a single class of differentially expressed genes. The top 20 over-represented pathways from the meta-analyses of over-represented pathways are shown in Table 2.

**Table 2:** The top twenty over-represented pathways determined by the meta-analysis combining the GWAS and the gene expression PD studies.

GWAS and Gene Expression	Meta_P-value	Corrected P-values
Axon guidance	1.39E-09	2.79E-07
Focal adhesion	3.82E-08	7.68E-06
Calcium signaling pathway	1.42E-07	2.85E-05
Cell adhesion molecules (CAMs)	1.04E-06	2.09E-04
Adherens junction	1.18E-06	2.37E-04
Renal cell carcinoma	2.70E-06	5.43E-04
Regulation of actin cytoskeleton	1.35E-05	2.71E-03
Non-small cell lung cancer	8.40E-05	1.69E-02
Melanogenesis	9.59E-05	1.93E-02
ErbB signaling pathway	1.15E-04	2.31E-02
Long-term potentiation	2.62E-04	5.27E-02
Glioma	2.71E-04	5.44E-02
Type I diabetes mellitus	3.53E-04	7.10E-02
Pancreatic cancer	6.28E-04	1.26E-01
Phosphatidylinositol signaling system	6.98E-04	1.40E-01
Neuroactive ligand-receptor interaction	1.31E-03	2.63E-01
Complement and coagulation cascades	1.39E-03	2.79E-01
Heparan sulfate biosynthesis	1.62E-03	3.25E-01
MAPK signaling pathway	1.71E-03	3.43E-01
Endometrial cancer	2.86E-03	5.75E-01

The over-represented pathways are presented in rank order.

## Discussion

The pathways identified by integrating GWAS and gene expression data reported here have both statistical and biological support to be involved in PD. This further suggests that many of the changes seen in gene expression studies are not due to secondary, non-specific changes, but rather have direct influence on the pathogenesis of the disease. It also demonstrates that the pathways derived from the GWAS have biological support. The convergence of these two greatly reduces the risk that these pathways are biased by size of the significant gene, with larger genes having greater chance of having a significant SNP [14]. O'Dushlaine [15] examined the effect of the number of genes in a pathway towards significance, but didn't see any correlation. Supporting this are recent studies of pathway analyses in other disorders such as Diabetes and Inflammatory diseases [14], [16], where little overlap was seen between these pathways and those reported here. Had the size of the pathway been a prominent factor, more overlap would be expected. Elbers *et al.* [14] examined this possibility as well, but only found it to be a problem with two of the pathway classification tools they tested, neither which was utilized in this analysis.

Previously, a SNP ratio test (SRT) has been developed to assess enrichment of significant associations from GWAS in context of KEGG pathways [15]. One difference between the SRT method [15] and the method described here is with respect to the mapping of SNPs to genes. In our study, the mappings of SNPs to genes are obtained using a window of 20 Kbp on either side of the SNP. The genes identified within the window are added to the gene list of interest. Using the criterion, if no gene was identified for the SNP in question then the two neighboring genes, irrespective of distance separation between the SNP and gene, are added to the gene list of interest. In the O'Dushlaine et al. study [15], the mappings of SNPs to genes are obtained by parsing the db SNP table b129_SNPContigLocusid_36_3.bcp to include SNPs less than 2 Kbp from the 5′ of the gene and less than 0.5 Kbp 3′ from the end of the gene.

One reason we chose our method was that variants regulating transcript expression or transcriptional control are likely to be important in complex disease, and to date many associated SNPs in these disorders have been located in “gene deserts” or non-coding regions. While there have been no systematic studies to investigate the affect of upstream and downstream analysis for the cause and the effect of genetic variation, one study has shown that 95% of genetic variation affecting transcript levels is within 20 Kbp of the transcript start and end sites [17] and therefore provides support for our use of 20 Kbp radii for mapping SNPs to genes. Furthermore, it is likely that SNPs located in gene deserts may coincide with enhancers and silencers, thereby providing a rationale to search beyond the 20 Kbp to attach a SNP to gene when protein coding genes are absent within the initial 20 Kbp search. Studies using conserved non-coding elements (CNEs) and in-vivo GFP enhancer assays have shown that the majority of CNEs are located in gene deserts and are likely to up-regulate effector genes of up to 250 Kbp away. Many of the CNEs have a larger distance of separation from the target genes (>1 Mbp) [18]–[22]. Targets for these enhancers are enriched for the regulation of transcriptional control or developmental genes [19], [20], many of which have been identified in these pathways. It is likely the methods used here to map SNPs to genes will be valuable to establish evolutionary significance of long range cis-regulation and trans-regulation in complex diseases [17], [20], [22]–[26].

We believe that the measurement of gene expression compared between different CNS regions affected by PD within the same person helps adjust for factors such as agonal state, postmortem interval and other environmental effects that affect RNA quality. By using differences between anatomical regions from the same patient, we create an internal reference for these effects. Further, this methodology should be more sensitive to identifying expression changes in individuals at different stages of disease that would be “washed out” by the traditional analysis of groups of cases versus controls.

In this study, we used Fisher's combined probability test to combine the results of pathway analysis in a large GWAS dataset and a smaller expression dataset. One limitation of this approach is that it gives equal weight to the results from each dataset. While this does not seem to be a big concern in the current study given the concordant results in the two datasets, it may not be optimal for other studies attempting to follow a similar approach with more disparate results (and heterogeneous independent data sets). In such cases, weighting each dataset's contribution by a quality score, effect size, or variance on the test statistic might be desirable and can be accomplished by employing more complex methods that accommodate such weights.

**Figure 2:**
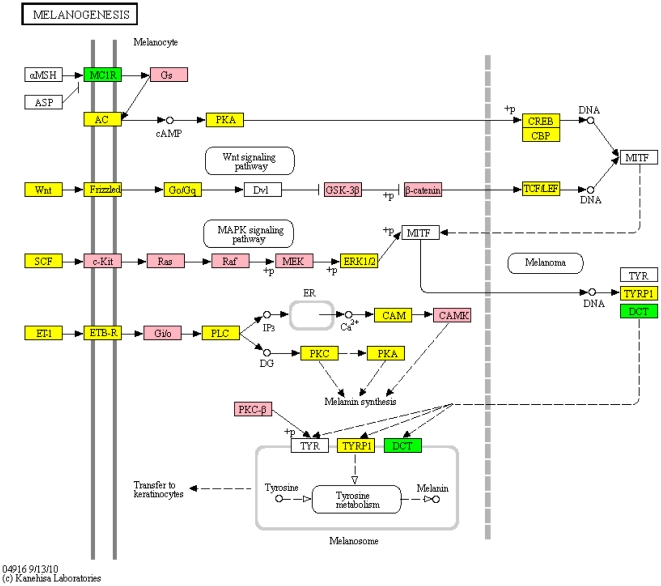
KEGG pathway for melanogenesis. Similar to a gene expression array, those significant genes in the GWAS study are green, those in the expression study are pink and those that are significant in both are yellow.

Both melanoma and melanogenesis pathways were identified in the GWAS data as significant pathways (Figure 1), and the melanogenesis pathway was ranked ninth in the meta-analysis (corrected p-value  = 1.9E-02). This seems biologically intuitive, as melanin, tyrosine and their biosynthesis (Figure 2) are tightly tied to the production of dopamine, the neurotransmitter deficient in PD. Interestingly, other investigators have suggested that common genetic determinants exist in the causal pathway to melanoma and PD [27]–[29]. It seems also intuitive that much of this pathway is lost in the gene expression analyses due to the destruction of substantia nigra, and therefore is not prominent in the gene expression results, though several genes in the pathway are found significant in the expression analysis.

What is striking is that none of the SNPs in the melanogenesis pathway (Figure 2) have been strongly implicated in current association studies. This is because no one SNP or gene in these pathways has met the stringent criteria of genome-wide statistical significance despite multiple GWAS studies. The idea of focusing on single gene or SNP effects when examining a disease is derived from the very successful research over the past 30 years on Mendelian (single gene) disorders. But, as seen here, the origin of complex disease is really that of the cumulative effect on genes functioning together in pathways. The data reported here support the thoughts of other authors that pathway analysis is the more appropriate analysis when looking for genes involved in these diseases [6]. We would suggest that pathway analysis becomes part of every approach to gene discovery in complex disease. It seems likely, based on the concordance seen here by the three GWAS studies in Edwards *et al*. [1] and the gene expression data, that pathway analyses are more likely to replicate across GWAS studies than single gene analyses.

It is interesting that the other well known Mendelian PD genes (SCNA, PARKIN, DJ1, and PINK1) are not directly included in any of the identified significant pathways. There are several potential reasons for this finding. It may reflect the lack of knowledge about the regular function of these genes. Or it is possible that Mendelian mutations leading to PD are unique genetic initiation points for PD, and while very insightful to the general mechanisms that can lead to PD, are not involved in directly in the pathways whose genetic variability contribute to susceptibility to “idiopathic” PD.

Axon guidance was one of the top over-represented pathways, and is important for brain development, dopaminergic axonal maintenance, regeneration and target recognition. In 2005, Maraganore *et al.* [30] published a tiered association study on PD and identified a SNP in semaphoring 5A (SEMA5A) as the most significant association. While this has not been replicated in subsequent studies, as SEMA5A is important in axon guidance, they examined SNPs in other genes in this pathway to attempt to utilize these SNPs to predict outcome in patients. They found support for additional axonal guidance pathway genes to be associated with PD, and using the dataset from Papapetropoulos *et al.* [11] found more differential expression than they expected for 45 genes in the axonal guidance pathway, using only data from the substantia nigra and striatum. Wang *et al.* [31] using a different, smaller GWAS dataset, also found it to be the only significant pathway. Sutherland *et al*. [32] did a meta-analysis of reports of gene expression studies in PD, excluding the data utilized here, and also found axon guidance to be one of the major pathways over-represented, as did Bossers *et al.* in a recent gene expression study [33].

Focal adhesions (FA) are specialized regions where the cell interacts with the extra cellular matrix (ECM). One primary function of FA is linking the actin cytoskeletal network to the transmembrane integrins (KEGG). Other FA are involved in signaling between the cell and the ECM, as well as helping regulate phosphorylation of molecules like MAPT, and influencing receptors for growth factors. Errors in the interaction between the ECM/FA can lead to a form of apoptosis called anoikis [34]. Caltagarone *et al.* [35] has suggested that errors in the FA/integrin signaling pathway, in response to oxidative stress and fibillary Aβ, is one of the key pathways in inducing cell death in Alzheimer disease (AD).

Adherens Junction (AJ) proteins are involved in the cell-cell adhesions. In tissues such as skin, they are involved in maintaining cell contact during external stress such as movement [36]. Cadherins are core AJ proteins and interact with microtubules and actin filaments. In the brain, AJ proteins are involved in maintaining the blood-brain barrier (BBB). Interestingly, one of the primary regulators of AJ in the BBB is calcium [37]. Changes in BBB have been reported in normal aging [38], and several studies have suggested changes in BBB exist in PD patients [39], [40].

**Figure 3:**
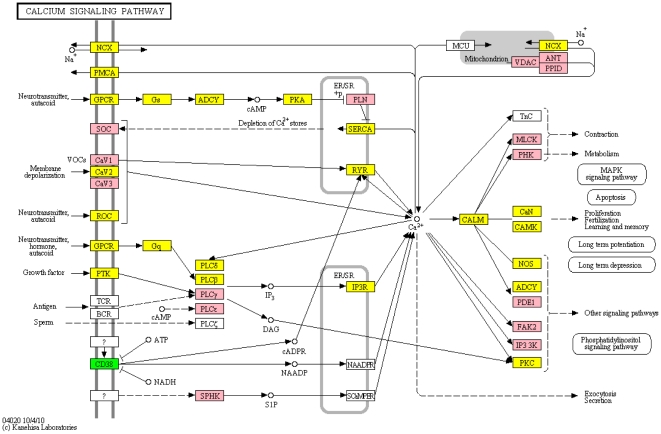
KEGG pathway for calcium signaling. Similar to a gene expression array, those genes with significant expression in the GWAS study are green, those in the expression study are pink and those that are significant in both are yellow.

The calcium signaling pathway (Figure 3) has an important role in regulating a great variety of neuronal processes, as mentioned above. Its high rank in the meta-analysis fits well with the recent work that has shown the SNGRA dopamine neurons have an unusual reliance on voltage-dependent L-type Ca2+ channels in autonomous pace-making. This suggests that the mitochondrial stress created by sustained Ca2+ entry could be responsible for their selective vulnerability, rather than simply a late stage consequence [41], [42]. This hypothesis is also consistent with the centrality of mitochondria in prevailing models of pathogenesis in PD, as it serves as one of the primary sources of Ca++ buffering. Deficiency of one known PD gene, PINK1, has been reported to cause mitochondrial accumulation of calcium, resulting in mitochondrial calcium overload [43].

Recently, Satake *et al*. [44] reported an association reaching genome-wide significance of BST1 with PD in a Japanese population. Interestingly, BST1 (CD157) is closely related to CD38, which is one of the genes in the calcium pathway, and did demonstrate significance in our GWAS data as well (Figure 3). These two ectoenzymes and surface receptors utilize NAD+ and are located within 50 kb of each other on 4p15. It is possible that a common variation affecting both genes is in linkage disequilibrium with the different SNPs in each population.

**Figure 4:**
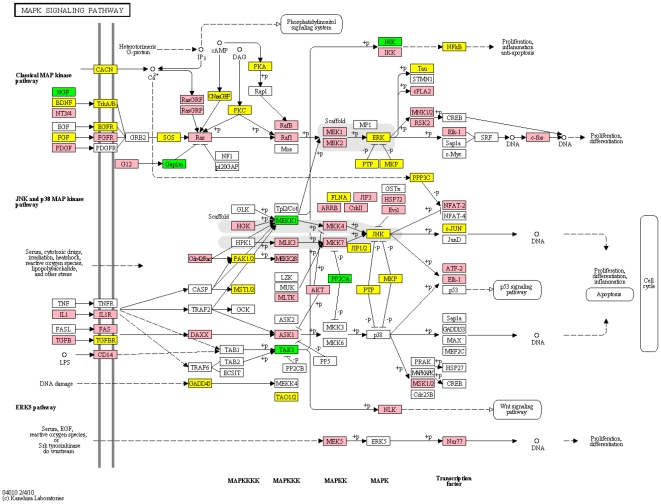
KEGG pathway for MAPK. Similar to a gene expression array, those genes that are significant in the GWAS study are green, those in the expression study are pink and those that are significant in both are yellow.

**Figure 5:**
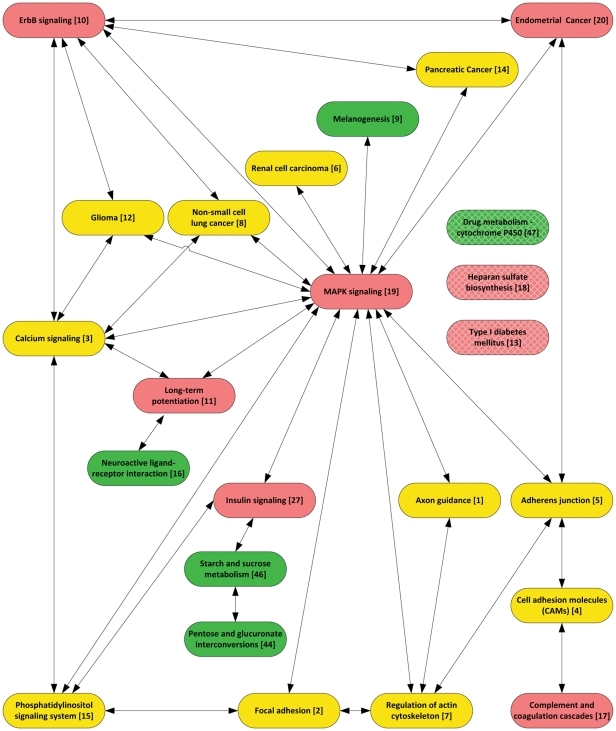
Cross-talk amongst over-represented pathways in PD. The links are defined by the LinkDB function from the KEGG website. The top ten over-represented pathways from the GWAS and expression studies (Table 1) plus the top twenty over-represented pathways from the meta-analysis (Table 2) are shown. The insulin signaling pathway, linking the MAPK signaling with the starch and sucrose metabolism, is also shown. Three pathways (drug metabolism - cytochrome P450, heparan sulfate biosynthesis and type 1 diabetes mellitus shown with gray thatched background) did not cross talk with the other top pathways over-represented in this study. The pathway's rank in the meta-analysis is provided in parenthesis. The pathways unique to the GWAS PD study are green, those unique to the expression PD study are pink and the pathways common to both expression and GWAS PD studies are yellow.

The mitogen-activated protein kinases (MAPKs) are a major set of metabolic reactions and thus it is not too surprising they appear to play a central role in biological cross-talk of the various identified pathways (Figures 4 & 5). MAPKs are serine-threonine kinases that mediate intracellular signaling associated with a host of cellular activities that include cell proliferation, differentiation, survival, death and transformation [45]. Mammals express at least four distinctly regulated groups of MAPKs: the extracellular signal-related kinases (ERK)-1/2, c-Jun N-terminal kinases (JNK1/2/3), p38 proteins (p38 alpha/beta/gamma/delta) and ERK5. The MAPT gene (tau) is one of the most replicated associations with risk for PD, and is in the MAPK pathway which is activated by ERK (MAPK1). Both JNK and ERK have been shown to contribute directly to mitochondrial dysfunction by suppressing oxidative respiration when activated by various models of PD [46]. Parkin has been reported to directly inhibit JNK activation via ubiquitination of JNK pathway mediators [47].

While PD is one of the most common movement disorders, the molecular mechanisms underlying neuronal degeneration in PD are still unclear [48]. By converging independent genomic datasets, we have identified pathways that have both biological function and genetic support to be important in PD. As pathways, they should be more replicable between datasets than single SNPs. Our data strongly supports the concept that complex diseases should be evaluated as pathway diseases and that the failure of GWAS studies to identify and replicate significant SNPs may reflect the conceptual failure of the genetics field to move beyond single-SNP analytic strategies.

Most of the pathways identified here have not been extensively studied in PD to date. It is interesting that the pathways from the meta-analysis suggest that it is the genes involved in the cells signaling and structural interactions with its environment whose genetic variation seems to have the most effect on susceptibility to PD. While it could be argued that the identification of these pathways are secondary changes due to cell loss, the convergence of the GWAS data on the same pathways argues strongly against this. This seems to lead one in a different direction than much of the current PD research based on specific single gene mutations. These pathways are important to how the cells react to stress and one another, and are also important in developmental aspects of the CNS as well. They raise the interesting concept that perhaps all of us are exposed to the initiating events or stresses that give rise to PD, and it is the cumulative genetic makeup of these and other similar pathways in each individual that strongly contributes to whether we develop PD or not.

## Materials and Methods

### Gene Expression Samples

**Figure 6:**
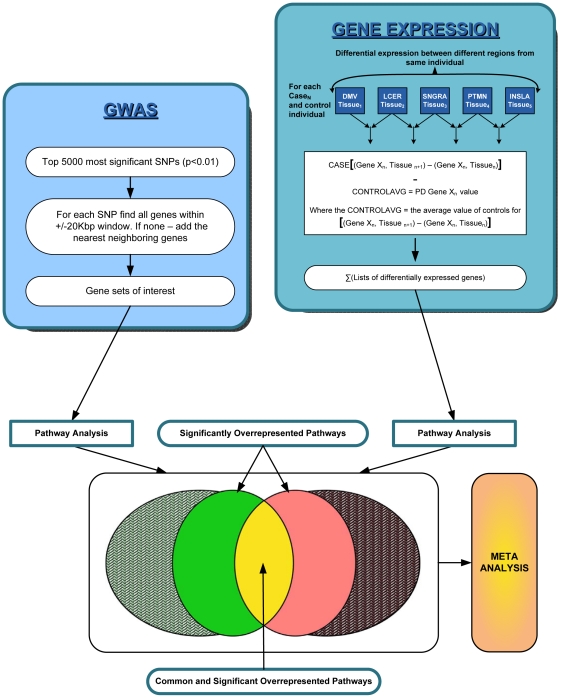
Conversion of Gene Expression and GWAS Pathways. For gene expression analysis, differentially expressed genes between adjacent tissues affected in PD were identified in each individual and adjusted using average control expression profiles. Three subtractions were used to identify genes of interest in PD. The first subtraction between two adjacent tissues relates to a given PD patient (eg CASE N). The second subtraction is in controls, where a similar subtraction as above is performed, and then averaged over the four controls for the two given adjacent tissues. This is denoted as CONTROLAVG. To establish if gene X is of interest in PD between two adjacent tissues, the CONTROLAVG was subtracted from the first subtraction for each patient. An overview of the GWAS PD study is shown. Pathway analysis was performed on each of the GWAS and gene expression PD gene sets derived. Two types of convergence were performed. First, significantly over represented pathways common in both studies were identified (Figure 1). Second, a meta-analysis was carried out to combine the results obtained from the gene expression and the GWAS PD pathway analyses (Table 2). DMV = dorsal motor nucleus, LCER =  locus ceruleus, SNGRA =  substantia nigra, PTMN =  putamen, INSLA =  insula.

For the gene expression analysis, we utilized the Affymetrix Plus 2.0 microarray data set of Papapetropoulos *et al.* [11]. In this study, 22 PD patients and 23 controls were collected through the University of Miami Brain Bank (D. C. Mash, Director). Twenty-one different brain regions from each individual were isolated and gene expression was performed. All patients met UK PD Society Brain Bank diagnostic criteria and controls were clinically normal and had no evidence of neurodegeneration upon autopsy. The average age of controls was 78 years; PD cases were 74 years. The six anatomical regions used by Braak et al to determine their neuropathic stages of PD [49] provide a convenient method for identifying multiple anatomical regions of the brain involved in the neurodegeneration of PD. To perform our analysis, we required all patients to have the same anatomical regions available for the analysis. But Papapetropoulos *et al.* did not perform gene expression analysis on many of the 21 anatomical brain regions isolated per person due to poor quality mRNA of those specific tissues. Thus, we sorted the raw gene expression data per patient per brain region to maximize the availability of samples for our analysis. No individuals had gene expression data available on tissue lying in all six "Braak" regions. Seven patients and four controls had gene expression data on five of the six anatomical regions and these were used for the analysis. The five brain regions representing the Braak stages were: the dorsal IX/X motor nucleus (DMV) for Stage 1, locus ceruleus (LCER) for Stage 2, substantia nigra (SNGRA) for Stage 3, putamen (PTMN) for Stage 4, and the insula (INSLA) for Stage 5. The general outline of the methodology is shown in Figure 6. As shown, the differential expression was measured between anatomically adjacent tissues within the same individual first. Thus, subtraction LCER vs. DMV was calculated by taking the expression value of each gene in DMV and subtracting it from the expression value in LCER. The process was repeated for each stage, ending up with four subtractions per individual: LCER vs. DMV, SNGRA vs. LCER, PTMN vs. SNGRA, and INSLA vs. PTMN.

### Gene Expression Analysis

The raw data were normalized using the GCRMA algorithm in GeneSpring (Agilent Technologies, Santa Barbara, CA). Statistical tests, fold change calculations, hierarchical clustering of the data, and annotations were all performed in GeneSpring GX7.3.1. The data were then imported to Excel for further manipulations.

For each patient or control individual, genes that were significantly differentially expressed with a Benjamini q-value of <0.05 [50] were then taken into a subtraction analysis. Ratios were then selected at each stage with respect to the stage previous to it. Thus, subtraction LCER vs. DMV was calculated by taking the expression value of each gene in DMV and subtracting it from the expression value in LCER. The subtractions were performed using data manipulation tools in Excel. The process was repeated for each stage, ending up with four subtractions: LCER vs. DMV, SNGRA vs. LCER, PTMN vs. SNGRA, and INSLA vs. PTMN. The four controls were treated as replicates and the average expression profile for each subtraction of the control data was calculated. This analysis was done to allow us to discount the control expression profiles from the patient data, without making any assumptions as to which controls would be matched to which patients. The average control expression profile per brain region was then subtracted from the corresponding brain region expression profile of each patient (Figure 6). Statistically-determined differentially expressed genes (Benjamini p-value of <0.05) at each transitional stage were selected for further analysis. Within each subtraction, genes of values (fold change differential) ≥1.6 are considered to have been up regulated between the two brain regions. Similarly, if the differential value was ≤−1.6, that gene was down regulated in the later Braak stage than in the earlier Braak stage. Note that since there is no replication per se, each individual is unique and each brain region is unique per individual, using standard t-tests or one-way ANOVAs for comparison of gene expression is not applicable to this data set. The lists of differentially expressed genes were generated and passed through GOstats for pathway analysis.

### GWAS sample Analysis: Mapping SNPs to genes

We used data from a previous study [1], which is a joint analysis of three PD GWAS datasets imputed up to the Illumina 610 genotyping chip (1752 PD cases and 1745 controls). To focus on the most significant pathways, 5,000 SNPs with the most significant association (p<0.01) were used in this analysis. To map SNPs to genes, we used the chromosome and position of the SNP, and located the genes within a window 20 Kbp upstream and downstream of the SNP. The annotations for mapping the SNPs to the genes were from dbSNP version 129 and the ENSEMBL database (homo_sapiens_variation_50_361; homo_sapiens_core_50_361; homo_sapiens_registry_50_361) [51]. The annotations for human genome assembly version 36.1 were used. If these top SNPs contained multiple SNPs for the same gene, the gene was only counted once, so linkage disequilibrium would not be a factor. If there were no genes found in a+/−20 Kbp window, the closest gene on each side of the SNP were both included. A PERL script (snps2Genes.pl) was developed and used to perform the above analysis. A query list (comprising 2619 genes) for pathway analysis was derived from the top 5000 SNPs. The reference gene list was obtained from the joint analysis dataset [1] with combined sample generated genotypes at 495,715 SNPs after imputation (and sample SNP quality control filters). These SNPs were used as the reference data set and the SNP to gene mapping protocol was used to map SNPs to genes; 18,810 unique genes were established as the reference gene list. Only protein coding genes were considered for the query and reference.

### Pathway Analysis

GOstats [52], a Bioconductor package written in R, was used to examine the pathways in KEGG [53]–[55]. The org.HS.eg.db package was used to assign KEGG pathway annotations to Entrez gene identifiers. Over-represented pathways for given gene lists were calculated using a classical hyper-geometric statistical comparison of a query gene list against a reference gene list using GOstats. The GOstats method was applied to the expression and the GWAS data independently. For the pathway analysis of expression data, the query gene lists are the differentially expressed genes. The differentially expressed genes were used to compare against a reference set of 21,218 genes on the microarray chip used in Papapetropoulos *et al.* (hgu133plus2). Up-regulated genes were considered separately from down regulated genes unless otherwise stated. For the pathway analysis of GWAS data, the query gene list was derived from SNPs with p-values ≤0.01, (5000 SNPs) and the reference gene list was obtained from the GWAS (see previous section). A PERL script (genes2ORPathways.pl) was developed and used to search for over-represented pathways in query gene lists relative to the reference gene lists. While there are 343 pathways currently in KEGG pathway database only 205 were relevant to the human genome [55].

### Meta-analysis of over-represented pathways

A meta-analysis of the pathway results from GOstats was performed using the Fisher's combined probability test. As the method requires independent tests, we did the meta-analysis on the GWAS pathway analysis and a pathway analysis based on a gene list of differentially expressed genes. P-values are combined by adding the -2ln (p-value) for the two tests for a pathway. This value follows a chi-square distribution which was used to determine the combined p-value.
